# Effect of Lecithin on the Spontaneous Crystallization of Enzymatically Synthesized Short-Chain Amylose Molecules into Spherical Microparticles

**DOI:** 10.3390/polym11020264

**Published:** 2019-02-04

**Authors:** Carlos Andres Morales Letona, Ke Luo, Ki-Baek Jeong, Hazzel Joy Adra, Cheon-Seok Park, Young-Rok Kim

**Affiliations:** Institute of Life Sciences and Resources & Department of Food Science and Biotechnology, College of Life Sciences, Kyung Hee University, Yongin 17104, Korea; andressagara@gmail.com (C.A.M.L.); nageuk@khu.ac.kr (K.L.); nabluedust@naver.com (K.-B.J.); hazeadra@gmail.com (H.J.A.); cspark@khu.ac.kr (C.-S.P.)

**Keywords:** polymeric microparticles, amylosucrase, amylose, self-assembly, lecithin, uniform, amylose microparticles

## Abstract

Here, we report a facile and effective one-pot approach to prepare uniform amylose-based polymeric microparticles (PMPs) through enzymatic synthesis of short-chain amylose (SCA) followed by spontaneous self-assembly of the SCA in the presence of lecithin. The effect of lecithin on nucleation and growth kinetics of amylose microparticles was investigated by monitoring the turbidity of reaction solution and the size of particles over the course of the self-assembly process. The results suggest that lecithin played a critical role in controlling the self-assembly kinetics to form uniform amylose microparticles through steric stabilization of the growing particles and diffusion-limited growth effect. The crystallinity of amylose microparticles was not affected by lecithin, implying that lecithin did not disrupt the crystal structure within the particle and would mainly be present on the surface of the microparticles. Considering its biodegradable and biocompatible nature, the amylose-based microparticles would find a range of useful applications in the area of food, cosmetics, medicine, chromatography and other related materials sciences.

## 1. Introduction

Amylose-based microparticles (AMPs), as biodegradable, biocompatible, and food-grade polymeric microparticles (PMPs), have emerged as a promising material for the development of functional microparticles, which is expected to have a number of potential applications in the field of food, cosmetics, chromatography [1], biosensors [2,3,4], and smart drug delivery [5]. Recently, crystallization of amylose, the mostly linear homopolymer of glucose linked with α-(1,4) glycosidic bonds, has become a useful tool for the preparation of amylose-based microparticles. It has been reported that short chain amylose (SCA, DP ≈ 40–50) can be synthesized by enzymatic polymerization using amylosucrase (DgAS) from *Deinococcus geothermalis* and sucrose as a sole substrate [6]. With its intrinsic nature of crystallization through hydrogen bonding and hydrophobic interaction, SCA can spontaneously be self-assembled into spherical microstructure in aqueous solutions without the need of organic solvents or energy consumption. However, the amylose-based PMPs are typically produced as an aggregated form with highly heterogeneous size distribution, which limits its practical applications. Therefore, an effective way of preventing such an aggregation or agglomeration during the synthesis of amylose microparticles remains to be resolved. 

As self-assembly progresses toward a supramolecular level, the fate of synthesized colloidal particles is heavily influenced by several factors, such as van der Waals (vdW) interactions, electrostatic double-layer interactions, and steric stabilization [7]. For example, the surface charge of particles as well as the pH of surrounding solution plays a key role in preventing the aggregation phenomenon between growing particles [8]. An appropriate steric stabilizer or surfactant could enhance the dispersion and stabilization of colloidal particles during the self-assembly reaction, where a frequent contact of particles and subsequent adhesion events by vdW interaction are hindered through their favorable interaction with the surrounding aqueous solution and steric stabilization effect on the surface of growing particles [8,9]. Here, we employed the stabilization property of lecithin to control the nucleation characteristics of SCAs and produced well dispersed amylose microparticles that are homogeneous in size and shape. Lecithin is typically originated from various food materials, such as eggs, soybeans, and milk, so it is widely used in the food industry as a safe food additive and dietary supplement. Thus, the advantages of amylose microparticles as biocompatible, biodegradable and as a food-grade material would not be deteriorated by the presence of lecithin in self-assembly reaction, and will be fully compatible with many applications in the food and biomedical area. 

Here, we present a simple one-pot synthesis of uniform AMPs through crystallization of SCA obtained enzymatically from sucrose as the sole substrate. The effect of lecithin on the growth kinetics and physicochemical properties of AMPs was investigated by turbidity analysis, scanning electron microscopy (SEM), Fourier-transform infrared (FT-IR) spectroscopy, X-ray diffraction (XRD) and differential scanning calorimetry (DSC) analysis.

## 2. Materials and Methods 

### 2.1. Materials

Sucrose, tris(hydroxymethyl)aminomethane hydrochloride (Tris-HCl), soybean lecithin (L-α-phosphadidylcholine ≥30%, P3644), imidazole, ampicillin, sodium chloride (NaCl), and monosodium phosphate (NaH_2_PO_4_) were purchased from Sigma-Aldrich (St. Louis, MO, USA). Luria-Bertani (LB) broth was obtained from Becton, Dickinson and Company (Franklin Lakes, NJ, USA). Ni-NTA Superflow resin was acquired from Qiagen Inc. (Valencia, CA, USA). 

### 2.2. Purification of Recombinant Amylosucrase from Deinococcus Geothermalis

The recombinant amylosucrase from *Deinococcus geothermalis* (DgAS) was prepared as described elsewhere [10]. Briefly, *Escherichia coli* MC106 harboring *dgas* gene in pHCE vector (pHCDGAS) was grown in 500 mL LB media containing 0.1 mg/mL ampicillin at shaking incubator (37 °C, 250 rpm) for 24 h. The cells were harvested by centrifugation (7000 xg for 20 min at 4 °C) and washed with a lysis buffer (50 mM NaH_2_PO_4_, 300 mM NaCl and 10 mM imidazole, pH 8.0). The bacterial cells re-suspended in the lysis buffer were disrupted by probe sonication (Sonifier 450, Branson, Danbury, CT, USA) in an ice bath. The cellular debris was removed by centrifugation (10,000 xg for 20 min at 4 °C), and the supernatant passed through a column packed with Ni-NTA resin (Qiagen Inc., Valencia, CA, USA). The Ni-NTA affinity column was washed with washing buffer (50 mM NaH_2_PO_4_, 300 mM NaCl and 20 mM imidazole pH 8.0) and the recombinant DgAS was eluted with elution buffer (50 mM NaH_2_PO_4_, 300 mM NaCl and 250 mM imidazole pH 8.0). The eluent containing DgAS was dialyzed to remove the excess imidazole and the buffer was replaced with PBS. 

### 2.3. Preparation and Characterization of AMPs and L-AMPs

The linear short chain amylose (SCA) molecules were synthesized by enzymatic polymerization using DgAS as described earlier [10]. Briefly, an aqueous solution containing 500 U DgAS and 500 mM sucrose in 1 mL of 50 mM Tris-HCl buffer (pH 7.0) was incubated at 30 °C for 24 h under constant rotation (15 rpm) using Rotator AG Model (FINEPCR, Gunpo, Korea). To investigate the effect of lecithin on the crystallization kinetics and morphology of resulting AMPs, varying concentrations of lecithin (0.01%-0.5%, w/v) was added to the polymerization reaction at 6 h from the beginning of reaction. Upon completion of the polymerization reaction, the reaction solution was incubated at 4 °C for another 24 h to induce crystallization of SCA into a particle form.

### 2.4. Characterization of the Size and Morphology of AMPs

The morphology of AMPs and L-AMPs were analyzed using a field emission scanning electron microscope (FE-SEM, Merlin, Carl Zeiss AG, Jena, Germany), operated at 3 kV. All samples were dehydrated in a vacuum desiccator before the analysis. The average sizes of AMPs and L-AMPs were determined by measuring at least 100 particles from the SEM images. The size growth of AMPs and L-AMPs over the course of the 24 h self-assembly reaction was monitored by using a dynamic light scattering spectrometer (Malvern Zetasizer Nano-ZS90, Malvern Instrument, Malvern, UK). The surface charge of AMPs and L-AMPs was measured using Dynamic light scattering (DLS, Zetasizer Nano ZS90, Malvern Instruments, Malvern, UK). 

### 2.5. Turbidity Measurement

Turbidity of the reaction was measured to monitor the rate of nucleation and growth of AMPs and L-AMPs. The absorbance of each reaction mixture was measured at 600 nm as a function of time for 24 h using a UV-Vis Spectrophotometer (Optizen POP, Mecasys Co. Ltd., Daejon, Korea).

### 2.6. X-Ray Diffraction (XRD)

The crystalline characteristic of each sample was determined using a diffractometer (D8 Advance, Bruker, Karlsruhe, Germany) equipped with Cu Kα radiation (0.154 nm). X-ray diffractograms were obtained with a generator voltage of 40 kV and current of 40 mA. Samples were scanned from 3° to 60° (2θ) with a step size of 6°/min.

## 3. Results and Discussion

**Figure 1:**
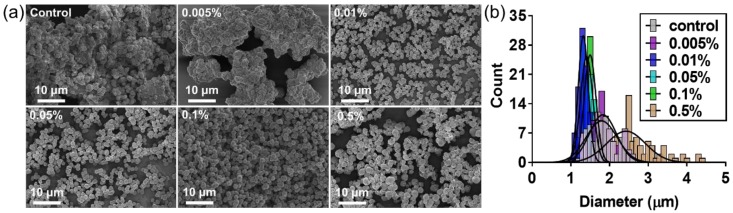
SEM image (**a**) and histogram of particle size distribution (**b**) of L-AMPs formed with varying concentration of lecithin from 0.005% to 0.5% (w/v). The amylose-based microparticles (AMPs) formed without lecithin were used as a control.

Short chain amylose (SCA) was obtained by the unique catalytic activity of DgAS, which hydrolyzes sucrose to fructose and glucose. The glucose was subsequently used as a first acceptor for successive glucosyl units that were generated from another hydrolysis reaction. Through the repeated cycle of hydrolysis and glycosidic linking, a homopolymer of α-(1,4) glucan chain was generated. In this study, lecithin was employed as a steric stabilizer to produce uniform AMPs, and the effect of lecithin on their morphology and particle size was investigated. SEM images clearly showed that uniform L-AMPs were produced at a given concentration of lecithin (Figure 1a). L-AMPs were shown to have a narrow size distribution with mean particle sizes of 1.0 μm to 1.5 μm (Figure 1b). The L-AMPs formed with 0.1 % (w/v) lecithin were found to have a mean diameter of 1.6 ± 0.2 um in an aqueous solution (Figure S1). The size of the same particles was measured to be 1.5 ± 0.1 um by SEM analysis, suggesting that the integrity of the particles, such as size and shape, was not significantly affected by the vacuum drying process. Based on the classic LaMer mechanism, uniform particles can be produced by a short homogeneous nucleation process that has minimum overlap with the particle growth phase [11]. The results suggest that lecithin could act as a steric surfactant to increase the surface charge density of SCA, generating sufficient electrostatic repulsive forces between the adjacent solid surface of SCA clusters to prevent their premature self-aggregation in an aggregated form during the nucleation stage (Figure S2). That is, the presence of lecithin is critical for the formation of uniform L-AMPs through an effective separation between nucleation and growth stages. On the other hand, the addition of lecithin at a concentration lower than 0.01% (w/v) had no effect on the size and morphology of AMPs, but generated AMPs in an aggregated form, similar to the microparticles formed without lecithin (control). A high concentration of lecithin (0.5%, w/v) was also not effective to induce the formation of uniform amylose microparticles. The particles formed with 0.5% (w/v) lecithin were shown to have a mean particle size of 2.4 μm, which is larger than control one (1.8 μm). In addition, there is no notable difference between the particle size and distribution of the particles formed at 4 °C and 30 °C (Figure S3). However, the particles formed at 4 °C were only included in this study since it has been reported that the recrystallization rate of starch-based microparticles was higher at 4 °C in comparison to 20 °C and 40 °C [12].

The L-AMPs formed with 0.5% (w/v) lecithin were shown to have a porous morphology (Figure 1a and Figure S4). It is likely that the lecithin molecules could form a micellar structure in reaction solution at a higher concentration and incorporate into the growing amylose microparticles. The lecithin micelles might be removed from the microparticles during the thorough washing step after the completion of the self-assembly reaction, leaving a porous structure in amylose microparticles. This assumption is supported by the critical micelle concentration (CMC) of lecithin in reaction condition. The turbidity of solution containing lecithin was measured as a function of concentration ranging from 0.0001 to 1% (w/v) (Figure S5). No changes in turbidity were observed when lecithin concentration was below 0.1%, but a sharp increase in turbidity was observed when the concentration was over 0.2% (w/v), suggesting that the CMC of lecithin is around 0.2%. A high concentration of lecithin was not effective in generating uniform and spherical microparticles, but the porous structure may find useful applications that require high surface area, such as chromatography, adsorbent, and food grade materials with enhanced digestibility. 

**Figure 2:**
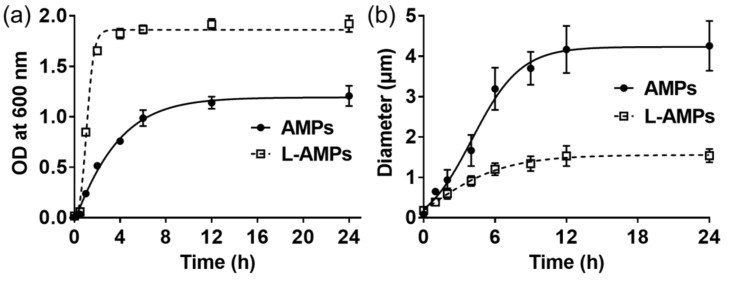
(**a**) Turbidity curves of reaction reflecting the nucleation and growth of AMPs and L-AMPs. (**b**) The growth of AMPs and L-AMPs over the course of 24 h self-assembly reaction at 4 °C. The L-AMPs were formed with 0.1% (w/v) lecithin.

In addition, we employed DNS (dinitro-salicylic acid) assay to determine the activity of amylosucrase used in the synthesis reaction. It was found that the activity of DgAS is not affected by the presence of 0.1% lecithin (Figure S6). The effect of lecithin on the nucleation and growth kinetics of AMPs was further investigated by monitoring the turbidity of reaction and the size of AMPs over the course of a 24 h self-assembly reaction (Figure 2). According to the absorbance measurement at 600 nm, the turbidity of reaction increased sharply for the first 2 h followed by a saturation for the rest of the self-assembly reaction when lecithin was present (Figure 2a). The turbidity of reaction reflects the nucleation and growth of AMPs [2,6,12]. The sharp increase of turbidity during the early stages of reaction indicates the fast formation of nuclei, which is essential for the formation of uniform particles. When the short nucleation process is accompanied by a quick reduction in the concentration of growth species below the minimum concentration of nucleation, further nucleation will not take place and the growth rate of all the nuclei will be the same, resulting in the formation of uniform-sized particles. However, the turbidity of reaction without lecithin increased slowly for the first 6–8 h. The slower rate of nucleation is likely to overlap with the growth phase and result in the generation of heterogeneous particles. The average size of AMPs increased to over 4 μm with a large standard deviation for the first 12 h, which is speculated to be caused by the heterogeneous nucleation and undesirable aggregation of growing particles during the growth phase (Figure 2b). The size of L-AMPs also increased during the early stage of reaction, but the particle size was saturated around 1.5 μm with a low standard deviation. The fast nucleation followed by growth phase would be responsible for the formation of uniform particles. The presence of lecithin on the surface of AMPs would also stabilize the uniform particles in an aqueous solution, which prevented the undesirable aggregation of growing particles. The soy lecithin used in this study is a mixture of three types of phospholipids, including phosphatidylcholine (PC, 29–46%), phosphatidylethanolamine (PE, 21–34%), and phosphatidylinositol (PI, 13–21%). PC and PE are zwitterionic surfactants that are electrically neutral at neutral pH. The highly negative charge density of lecithin (Figure S2) might be derived from the phosphatidylinositol (PI), which conferred negative charge on the surface of AMPs. The electrostatic repulsion in combination with the steric stabilization effect of lecithin played an important role in the formation of discrete and uniform particles. Considering diffusion-limited growth is desirable for the formation of uniform-sized particles, the lecithin on the surface of L-AMPs could also serve as an effective diffusion barrier to achieve diffusion-limited growth [13].

**Figure 3:**
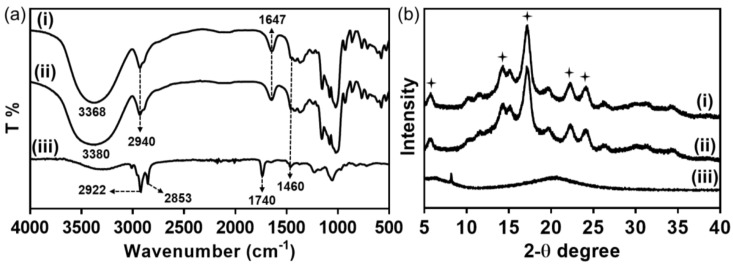
(**a**) FT-IR spectra and (**b**) XRD patterns of AMPs (i), L-AMPs formed with 0.1% (w/v) lecithin (ii), and pure lecithin (iii). The symbols (🟄) represent characteristic peaks for B-type crystals in AMPs.

FT-IR analysis was performed to investigate the interaction between lecithin and amylose molecules in L-AMPs (Figure 3a). Infrared spectrum of both AMPs and L-AMPs showed expanded OH stretching (4000–3300 cm^−1^). The peaks at 1647 cm^−1^ and 1460 cm^−1^ indicate the presence of bound water δ(H_2_O) in amylose microparticles and the C–H bending vibration, respectively [14]. Note that the absorption band derived from stretching vibrations of OH in L-AMPs was shifted to higher frequencies (3380 cm^−1^) with a broader peak when compared to that of AMPs (3368 cm^−1^). The shift of OH stretching and broadening of the peak would be attributed to the interaction of OH groups of phosphatidylinositol, as well as the phosphate groups present in lecithin with the OH groups of amylose molecules in amylose microparticles through hydrogen bonding. However, the characteristic peaks of lecithin, including 2922 cm^−1^ and 2853 cm^−1^ for C–H stretching and 1740 cm^−1^ for ester group, are absent in L-AMPs and only observed in pristine lecithin. This suggests that the amount of lecithin in L-AMP is too low to be identified by FTIR analysis and the trace amount of lecithin would mostly be present on the surface of AMPs. In addition, XRD analysis was carried out to investigate the effect of lecithin on the physical properties of the amylose microparticles. As shown in Figure 3b, both AMPs and L-AMPs had a typical B-type crystallinity with characteristic diffraction peaks at 5.6°, 15°, 17°, 22° and 24° [15]. This suggests that the presence of lecithin has a negligible effect on the crystallization of SCA in a hexagonal form, which is typical for B-type amylose crystals [16]. The B-type crystal nature of L-AMPs also precluded the possibility that the long hydrocarbon tails of lecithin might be buried within the cavity of amylose helix. Additionally, pure lecithin showed a broad diffraction peak at around 20°, which is indicative of the amorphous characteristics of lecithin [17]. It is speculated that lecithin is mostly present on the surface of L-AMPs, which did not disrupt the crystalline structure of amylose microparticles.

## 4. Conclusions

Uniform amylose microparticles were successfully prepared by amylosucrase-mediated synthesis of SCA and subsequent self-assembly of the SCA molecules in an aqueous solution. We employed lecithin as a steric stabilizer to control the self-assembly kinetics of amylose microparticles. The lecithin molecule introduced during the enzymatic synthesis reaction enhanced the nucleation rate for the self-assembly, leading to the homogeneous growth of nuclei to uniform particles. It is speculated that the enhanced nucleation rate of SCA molecules was caused by its complexation with lecithin during the synthesis reaction. The enhanced nucleation rate at the early stage of self-assembly reaction has been known to be effective in separating the nucleation phase from the growth phase. The separation of those two phases is essential for the generation of uniform particles. In other words, the formation of polydisperse particles is inevitable when the nucleation phase is overlapped with the growth one for a certain period of time, when nucleation and growth are simultaneously taking place. The negative charge of lecithin, mainly derived from phosphatidylinositol fraction, also confers negative surface charge on growing particles, which is strong enough to prevent undesirable inter-particle aggregations. The lecithin molecules on the surface of amylose microparticles also served as an effective diffusion barrier to control the transfer rate of growth species to the surface of particles. Combined, steric stabilization, enhanced nucleation rate and diffusion-limited growth effect were found to be responsible for the generation of amylose microparticles that are homogeneous in size and shape. The lecithin was shown to have a negligible effect on the crystallinity of amylose microparticles, suggesting that lecithin might be present on the surface of amylose microparticle without disrupting the crystal structure within the particles. Considering that both amylose and lecithin are biodegradable and biocompatible materials, this approach will find a range of applications in the fields of food, cosmetics, medicine and analytical sciences. 

## Supplementary Materials

The following are available online at http://www.mdpi.com/2073-4360/11/2/264/s1, Figure S1: Light transmission image and corresponding particle size distribution of L-AMPs formed with 0.1% lecithin, Figure S2: Zeta potential of lecithin, AMPs and L-AMPs formed with 0.1% lecithin, Figure S3: SEM Image of L-AMPs formed with 0.01% lecithin at 4 °C and 30 °C, Figure S4: SEM image of L-AMPs formed with 0.1% and 0.5 % (w/v) lecithin, Figure S5: Turbidity change of solution containing SCA and lecithin as a function of lecithin concentration, Figure S6: The concentration of fructose released from the synthesis reaction over the course of 24 h reaction.
