# Supplementary material for: Effect of Lecithin on the Spontaneous Crystallization of Enzymatically Synthesized Short-Chain Amylose Molecules into Spherical Microparticles
Source: Polymers (Basel). 2019 Feb 4;11(2):264. doi: 10.3390/polym11020264 (PMC6419240; doi:10.3390/polym11020264)
Supplement: Supplementary file 1 [file polymers-11-00264-s001.pdf]

Supplementary Data

Effect of lecithin on the spontaneous crystallization of enzymatically synthesized short-chain amylose molecules into a spherical microparticles

Carlos Andres Morales Letona, Ke Luo, Ki-Baek Jeong, Hazzel Joy Adra, Cheon-Seok Park, and Young-Rok Kim*

Institute of Life Sciences and Resources & Department of Food Science and Biotechnology, College of Life Sciences, Kyung Hee University, Yongin, 17104 South Korea

* Corresponding author.

Tel: +82-31-201-3830

Fax: +82-31-204-8116

E-mail address: youngkim@khu.ac.kr

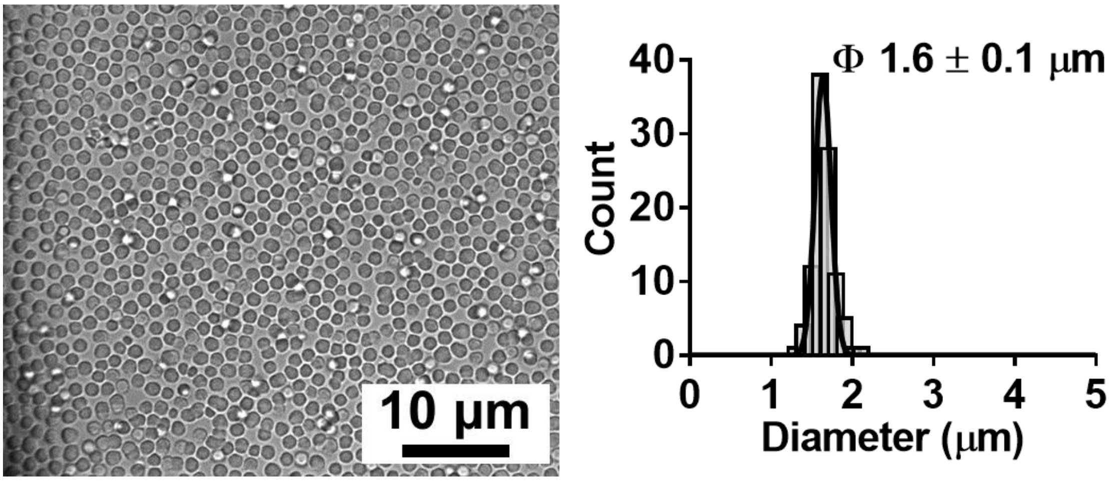

Figure S1. Light transmission microscopy image (left) and size distribution histogram (right) of L-AMPs formed with 0.1% lecithin.

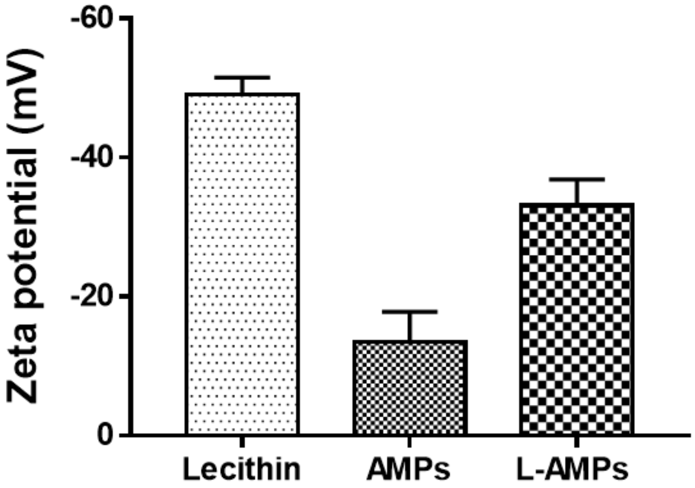

Figure S2. Zeta potential of lecithin, AMPs and L-AMPs formed with 0.1% lecithin.

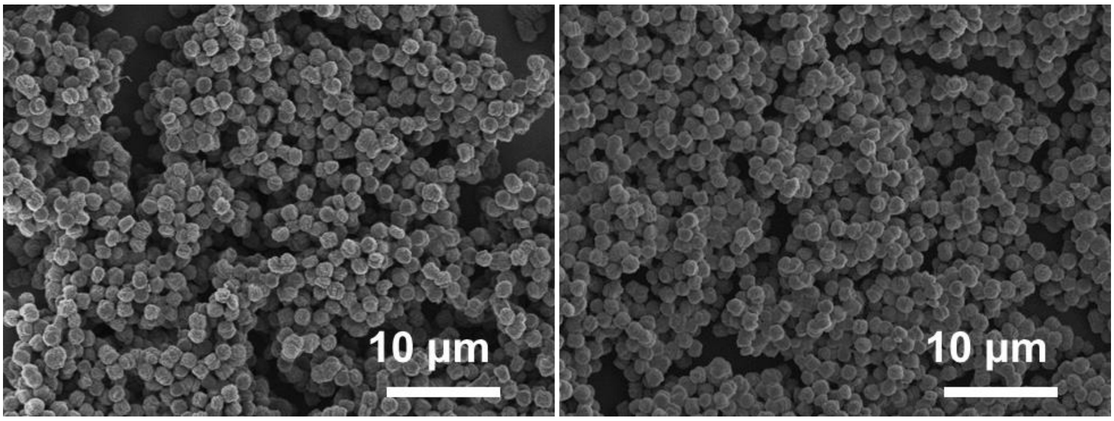

Figure S3. SEM Image of L-AMPs formed with 0.01% lecithin at 4 °C (left) and 30 °C (right).

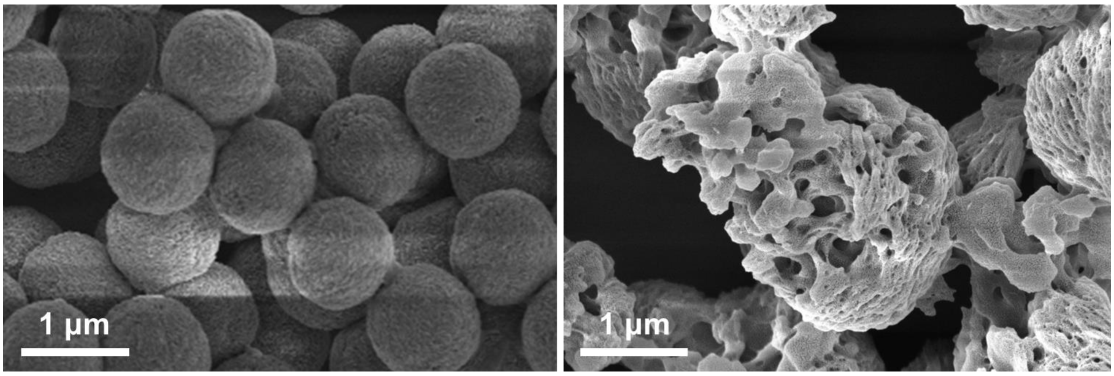

Figure S4. SEM image of L-AMPs formed with 0.1% and 0.5 % (w/v) lecithin.

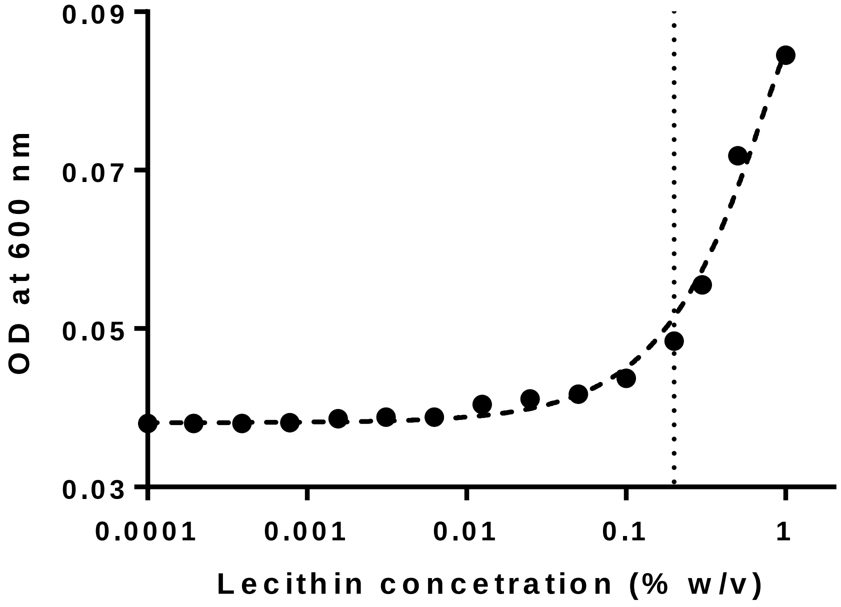

Figure S5. Turbidity change of solution containing SCA and lecithin as a function of lecithin concentration. The sharp increase of turbidity at lecithin concentration over 0.2 % (w/v) suggests that the critical micelle concentration (CMC) of lecithin is around 0.2 % (w/v) in given condition.

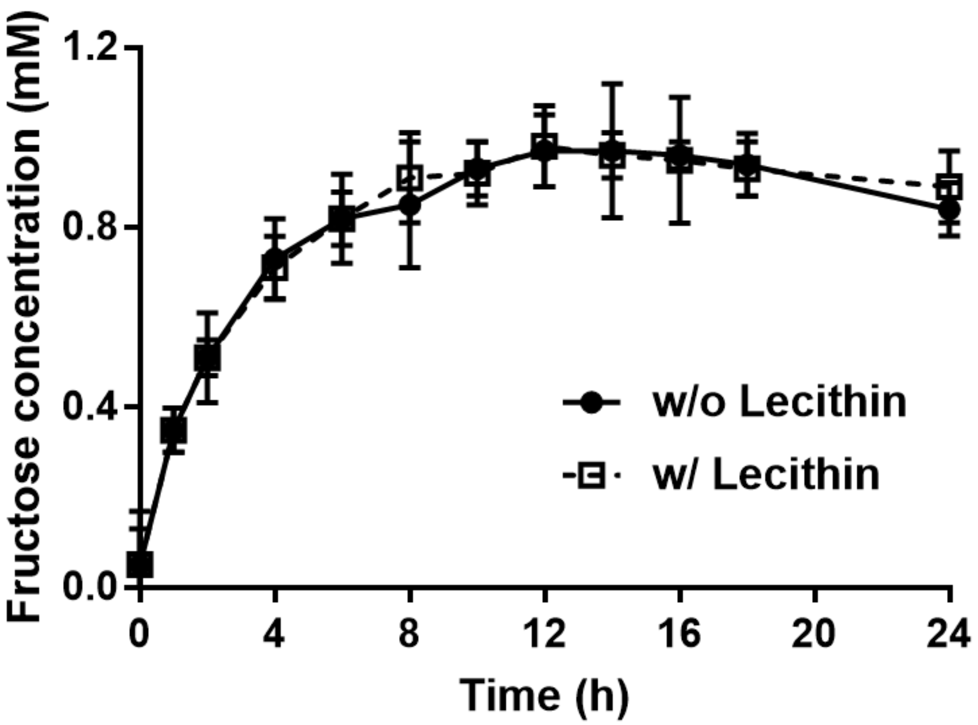

Figure S6. The concentration of fructose released from the synthesis reaction over the course of 24 h reaction. The activity of amylosucrase from *Deinococcus geothermalis* (DgAS) was not affected by the presence of lecithin. The enzyme activity of DgAS in the presence of lecithin was measured by determining its hydrolysis activity using dinitrosalicylic acid (DNS) assay as described elsewhere [1]. Briefly, a 1 mL of reaction mixture containing 500 U DgAS, 500 mM sucrose, and 0.1 % (w/v) lecithin in 25 mM Tris-HCl buffer (pH 7.0) was incubated at 30 °C, and 50 ul aliquot was collected at two-hour intervals over the course of 24 h reaction. The same reaction without lecithin was carried out as a control experiment. The samples taken at each time interval were mixed with 500 ul DNS solutions, followed by boiling for 5 min, and then stored in ice to avoid further color development. The O.D. of the reaction solution was measured at 575 nm using microplate spectrophotometer (Infinite M200, Tecan. Durham, NC, USA). A calibration curve using fructose as a standard was created to measure the concentration of fructose.

Reference

M.-C. Lim, K.-H. Park, J.-H. Choi, D.-H. Lee, C.A.M. Letona, M.-Y. Baik, C.-S. Park, Y.-R. Kim, Effect of short-chain fatty acids on the formation of amylose microparticles by amylosucrase, Carbohydr. Polym., 151 (2016) 606-613.
